# Patients with metabolic dysfunction–associated steatotic liver disease have preserved *in vitro* responses to antiplatelet drugs

**DOI:** 10.1016/j.rpth.2023.102217

**Published:** 2023-10-10

**Authors:** Bente P. van den Boom, André P. van Beek, Jelle Adelmeijer, Hans Blokzijl, Ton Lisman

**Affiliations:** 1 Surgical Research Laboratory and Section of Hepatobiliary Surgery and Liver Transplantation, Department of Surgery, University of Groningen, University Medical Center Groningen, Groningen, The Netherlands; 2 Department of Internal Medicine, Division of Endocrinology, University of Groningen, University Medical Center Groningen, Groningen, The Netherlands; 3 Department of Gastroenterology and Hepatology, University of Groningen, University Medical Center Groningen, Groningen, The Netherlands

**Keywords:** flow cytometry, hemostasis, nonalcoholic fatty liver disease, platelet aggregation inhibitors, platelet function tests

## Abstract

**Background:**

Patients with metabolic dysfunction–associated steatotic liver disease (MASLD) are at a risk of developing cardiovascular disease. Antiplatelet therapy not only prevents cardiovascular disease in these patients, but may also lower the risk of progression into advanced stages of fibrosis. However, patients with MASLD-associated cirrhosis often have complex changes in the hemostatic system and have been excluded from randomized trials.

**Objectives:**

The aim of this study was to assess the potency of antiplatelet drugs in these patients with MASLD-associated cirrhosis.

**Methods:**

We included patients with MASLD-associated cirrhosis (*n* = 19), patients with type 2 diabetes (DM2) and steatosis (*n* = 22), patients with steatosis only (*n* = 15), and healthy controls (*n* = 20). We measured basal platelet aggregation and activation using light transmission aggregometry and flow cytometry. We subsequently measured platelet aggregation and activation after *in vitro* addition of aspirin, cangrelor, and ticagrelor and compared the antiplatelet response in patients and healthy controls.

**Results:**

Rates of aspirin resistance as measured by light transmission aggregometry were similar between patients with MASLD-associated cirrhosis and healthy controls (21% vs 16%), but were significantly higher in patients with DM2 and steatosis (50% [*P* = .02] vs controls) and patients with steatosis only (53% [*P* = .05] vs controls). In patients with DM2 and steatosis, but not with MASLD-associated cirrhosis, the potency of cangrelor was significantly lower than that in healthy controls (*P* = .028).

**Conclusion:**

The *in vitro* potency of aspirin, cangrelor, and ticagrelor in samples of patients with MASLD-associated cirrhosis is similar to that of healthy controls. In contrast, the potency of commonly used antiplatelet drugs may be altered in patients with DM2 and steatosis and in patients with steatosis only.

## Essentials

• Antiplatelet drugs may have altered effects in patients with cirrhosis due to thrombocytopenia/thrombopathy.
• The potency of antiplatelet drugs was similar between patients with MASLD-associated cirrhosis and healthy controls.
• However, the potency of aspirin appeared lower in patients with DM2 and patients with steatosis.
• Further study is warranted to assess the relevance of the altered potency of antiplatelet drugs.

## 1 Introduction

Metabolic dysfunction–associated steatotic liver disease (MASLD) is the most common cause of liver disease worldwide and is regarded as the hepatic manifestation of the metabolic syndrome [1]. The term MASLD, previously known as nonalcoholic fatty liver disease (NAFLD), comprises a spectrum of pathologic entities [2], varying from the relatively benign presence of hepatic steatosis to the chronic inflammatory disease nonalcoholic steatohepatitis, which ultimately leads to cirrhosis.

Recent evidence has demonstrated that MASLD is not only associated with traditional cardiovascular risk factors such as type 2 diabetes mellitus (DM2) [3] and obesity [4], but is also an independent risk factor for the development of cardiovascular disease [5]. Antiplatelet therapy may thus be indicated to treat established cardiovascular disease in these patients. Interestingly, increasing evidence demonstrates that antiplatelet therapy may not only prevent cardiovascular disease [6,7], but may also result in lower risk of progression to advanced fibrosis in patients with MASLD [[8], [9], [10]]. Even though antiplatelet therapy might be indicated and potentially beneficial, its efficacy in patients with MASLD has yet to be determined.

Chronic liver disease, including MASLD, is associated with complex changes of the hemostatic system, such as decreased plasma levels of coagulation factors and proteins involved in fibrinolysis, and also thrombocytopenia and altered platelet function [11]. We have previously demonstrated altered *in vitro* and *in vivo* potency of anticoagulant drugs in patients with cirrhosis [12,13], but the capacity of antiplatelet drugs to effectively inhibit platelet function has not yet been studied in detail. Patients with liver disease have thus far been excluded from major randomized trials for antiplatelet drugs for the prevention or treatment of cardiovascular events. It is therefore unclear whether the dosages of clinically used antiplatelet drugs by the general population can be applied to patients with MASLD, specifically those with substantial thrombocytopenia. Importantly, growing clinical and laboratory evidence demonstrates that the antiplatelet effects of drugs such as aspirin and P2Y_12_ inhibitors vary greatly among the general population [14]. In some individuals, even a “resistance” to these drugs is described [[15], [16], [17], [18]], a phenomenon that remains poorly understood to this day. In this study, we aim to evaluate the *in vitro* potency of clinically used antiplatelet drugs in the blood of patients with various degrees of MASLD.

## 2 Methods

### 2.1 Study participants

This observational cross-sectional study was performed at the University Medical Center Groningen, The Netherlands, from January 2022 to July 2022. The study protocol was approved by the Medical Ethics Committee of the University Medical Center Groningen (METc 2021/411), and the study was conducted in accordance with the Declaration of Helsinki. Written informed consent was obtained from all participants enrolled in this study. Patients were enrolled from outpatient hepatology and diabetes clinics of the University Medical Center Groningen, The Netherlands, and healthy volunteers were included to determine reference values for the various tests performed. Patients were divided into 3 distinct patient groups: ie, patients with cirrhosis caused by MASLD, patients with a clinical diagnosis of DM2 and clinically relevant hepatic steatosis (as defined below), and patients without DM2 but with clinically relevant hepatic steatosis. The diagnosis of cirrhosis was confirmed by FibroScan liver stiffness results suggestive of F4 cirrhosis (liver stiffness > 13.6 kPa), histology compatible with Metavir F4 fibrosis, or radiologic features suggestive of cirrhosis. Historical clinical diagnoses were used to define the etiology of cirrhosis. Clinically relevant hepatic steatosis was defined by radiologic features suggestive of steatosis and/or FibroScan controlled attenuation parameter (CAP) results suggestive of S1 steatosis or higher (CAP > 302 dB/m) [19], and the absence of causes for secondary hepatic fat accumulation, such as significant alcohol consumption (>7 drinks per week for women and >14 drinks per week for men). Healthy volunteers were recruited using an informative poster in the University Medical Center Groningen, The Netherlands. Potential participants were screened for demographics, medical history, and use of medication. To prevent any bias that may be associated with age or sex, we aimed to include healthy volunteers within the same age ranges and proportions of males/females as those in our patient groups. Exclusion criteria for patients and healthy controls were an age of <18 years, evidence of malignancy, documented hereditary thrombophilia or hemophilia, HIV-positivity, pregnancy, use of anticoagulant medication (direct oral anticoagulants, vitamin K antagonists, and heparin), use of antiplatelet medication (aspirin and P2Y_12_ inhibitors), or use of nonsteroidal anti-inflammatory drugs (ibuprofen, naproxen, and diclofenac) within 3 days prior to inclusion. Additional exclusion criteria for healthy controls were history of liver disease or venous thromboembolism.

### 2.2 Analyses

All patients and controls underwent liver stiffness and CAP measurements using a FibroScan device. The antiplatelets drugs cangrelor (final concentrations: 0.5 and 0.125 μM; Sigma Aldrich), ticagrelor (final concentrations: 10 and 2.5 μM; Sigma Aldrich), or aspirin (final concentration: 100 μM; Genzyme Europe bv) were added to platelet-rich plasma samples or whole blood taken from patients and controls and incubated for 5 minutes at 37 °C prior to analyses using light transmission aggregometry in platelet-rich plasma and flow cytometry in whole blood. Experimental details are outlined in Supplementary Document 1.

### 2.3 Statistical analysis

Statistical analyses were performed using GraphPad Prism v9 and SPSS Statistics 28 (IBM). Data are expressed as median (with IQRs) or numbers (with percentages) as appropriate. Continuous variables were analyzed using the Kruskal–Wallis test (with Dunn’s post-test), and categorical variables were analyzed using the Chi-squared test. Spearman’s correlation coefficient was used to assess the association between continuous variables. Statistical significance was established at *P* < .05.

## 3 Results and Discussion

In total, we included 19 patients with MASLD-associated cirrhosis, 22 patients with DM2 and steatosis, 15 patients without DM2 but with steatosis, and 20 healthy controls. Of 19 patients with cirrhosis, 11 had an additional diagnosis of DM2. Demographic, clinical and laboratory data on inclusion are shown in the Table.

**Table:** Demographic, clinical, and laboratory data on inclusion.

Variables	Healthy controls (*n* = 20)	Cirrhosis (*n* = 19)	Type 2 diabetes with steatosis (*n* = 22)	Steatosis only (*n* = 15)
***Demographics***				
Age (y)	47 (41-58)	58 (54-67)^b^	52 (48-62)	47 (41-49)
Female (%)	13 (65)	11 (58)	14 (64)	8 (53)
White race (%)	20 (100)	18 (95)	21 (95)	14 (93)
Body mass index (kg/m^2^)	23.0 (20.6-24.2)	34.8 (28.6-36.8)^c^	34.6 (31.4-40.4)^c^	32.5 (29.9-35.5)^c^
Diabetic medication	n.a.			n.a.
Metformin		11 (58)	21 (95)	
Insulin		7 (37)	15 (68)	
Gliclazide		2 (11)	2 (9)	
Semaglutide		2 (11)	12 (55)	
Sitagliptin		1 (5)	0 (0)	
Cholesterol lowering agents	n.a.			
Statins		7 (37)	11 (50)	2 (13)
Ezetimibe		0 (0)	3 (14)	0 (0)
***Liver disease***				
FibroScan median score for liver stiffness (kPa)	5.0 (3.8-6.0)	28.9 (15.6-42.3)^c^	7.0 (5.8-8.3)^a^	5.9 (4.3-7.0)
FibroScan-controlled attenuation parameter (dB/m)	212 (178-238)	285 (241-324)^a^	323 (305-361)^c^	316 (269-350)^c^
MELD score	n.a.	8 (6-13)	n.a.	n.a.
Child–Pugh grade	n.a.		n.a.	n.a.
A		14 (74)		
B		4 (21)		
C		1 (5)		
***Hematology***				
White blood cell count (×10^9^/L)	5.5 (4.9-6.1)	5.2 (3.5-6.9)	8.5 (7.0-9.7)^c^	7.3 (5.8-8.7)^a^
Hemoglobin (g/L)	8.6 (8.1-9.1)	8.4 (7.5-8.9)	8.7 (7.9-9.2)	8.6 (8.3-9.6)
Platelet count (×10^9^/L)	244 (205-268)	109 (57-192)^b^	277 (201-351)	238 (200-277)
***Biochemistry***				
Urea (mmol/L)	5.0 (4.3-6.3)	4.5 (4.2-8.3)	5.1 (3.8-5.9)	5.0 (3.9-6.6)
Creatinin (μmol/L)	69 (61-81)	65 (58-87)	70 (57-78)	65 (62-84)
Bilirubin (μmol/L)	7 (6-10)	7 (4-23)	6 (3-7)	7 (3-10)
Gamma glutamyl transaminase (U/L)	15 (13-20)	88 (67-157)^c^	30 (23-58)^b^	39 (28-65)^b^
Aspartate transaminase (U/L)	23 (21-26)	40 (32-57)^c^	31 (22-44)	28 (26-52)^a^
Alanine transaminase (U/L)	17 (12-30)	35 (28-44)^b^	28 (21-56)^a^	33 (21-124)^b^
Lactate dehydrogenase (U/L)	177 (160-182)	208 (180-253)^b^	183 (159-219)	197 (183-217)
Alkaline phosphatase (U/L)	61 (47-69)	101 (84-138)^c^	90 (70-113)^c^	69 (66-76)
Glucose (mmol/L)	5.1 (4.8-5.3)	6.4 (5.9-8.7)^c^	7.9 (6.2-9.2)^c^	5.3 (5.1-5.7)
Total cholesterol (mmol/L)	4.6 (4.1-5.5)	3.6 (3.1-4.5)^a^	3.9 (3.3-4.6)^a^	4.6 (3.9-5.1)

MELD, Model for End-Stage Liver Disease; n.a., not applicable.

a *P* < .05 vs healthy controls.

b *P* < .01 vs healthy controls.

c *P* < .001 vs healthy controls.

Stimulation of platelets with TRAP-6 and AA resulted in a similar level of platelet aggregation in all patient groups compared to healthy controls, with the exception of patients with cirrhosis with a platelet count of <100 × 10^9^/L (Supplementary Figure S1). After stimulation with 2-MeSADP or XL-CRP, platelet aggregation in samples of patients with cirrhosis and in patients with DM2 and steatosis was significantly lower than that in samples of healthy controls. Platelet activation by all agonists was however comparable between groups when studied by flow cytometry, as evidenced by P-selectin or PAC-1 positivity (Supplementary Figure S2). No differences were observed between basal and agonist-induced platelet activation status analyzed by flow cytometry between patients with cirrhosis with a platelet count of below or above 100 × 10^9^/L (data not shown).

**Figure 1:**
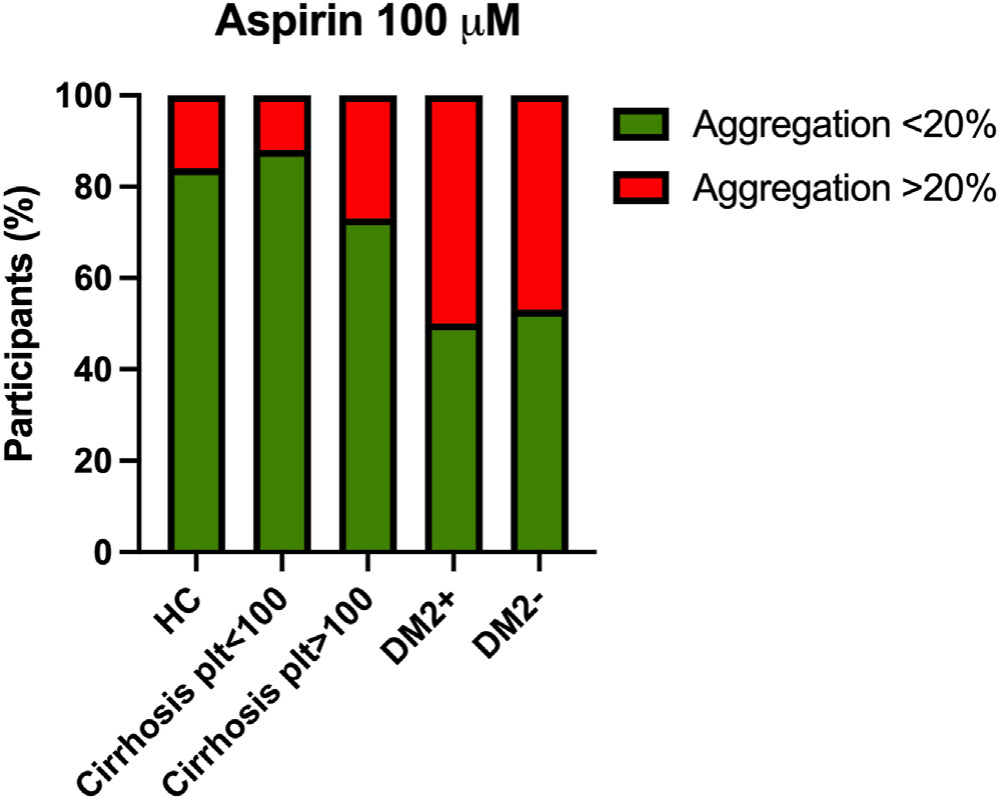
The effect of 100 μM of aspirin on the arachidonic acid–induced platelet aggregation at 6 minutes of light transmission aggregometry in HCs, patients with cirrhosis with platelet counts (plt) of below and above 100 × 10^9^/L, patients with type 2 diabetes and steatosis (DM2+), and patients with steatosis only (DM2-). Shown in the figure are the percentages of participants of which addition of aspirin to blood samples resulted in platelet aggregation of <20% (relative to platelet-poor plasma [100%] and platelet-rich plasma [0%]). HC, healthy control.

Given the lack of a detectable aspirin effect in flow cytometry analyses, we assessed platelet inhibition by aspirin only by light transmission aggregometry (LTA). In the untreated samples of 1 healthy control and 1 patient with cirrhosis with a platelet count of <100 × 10^9^/L, the platelets did not aggregate upon stimulation with AA. Therefore, these patients were excluded from analyses on aspirin resistance. In healthy controls, AA-induced platelet aggregation of >20% occurred in 3 of 19 (16%) samples treated with 100 μM of aspirin (Figure 1). The rate of aspirin resistance was similar in samples of patients with cirrhosis, independent of platelet count with a platelet aggregation of >20% in 1 of 8 (12%) samples of patients with a platelet count of <100 × 10^9^/L (*P* = .83 vs controls), and in 3 of 11 (27%) samples of patients with a platelet count of >100 × 10^9^/L (*P* = .45 vs controls). In samples of patients with DM2 and steatosis and of patients with steatosis only, rates of aspirin resistance were significantly higher than those in healthy controls, with 11 of 22 (50%, *P* = .021) and 8 of 15 (53%, *P* = .050) of samples with AA-induced platelet aggregation of >20%, respectively.

**Figure 2:**
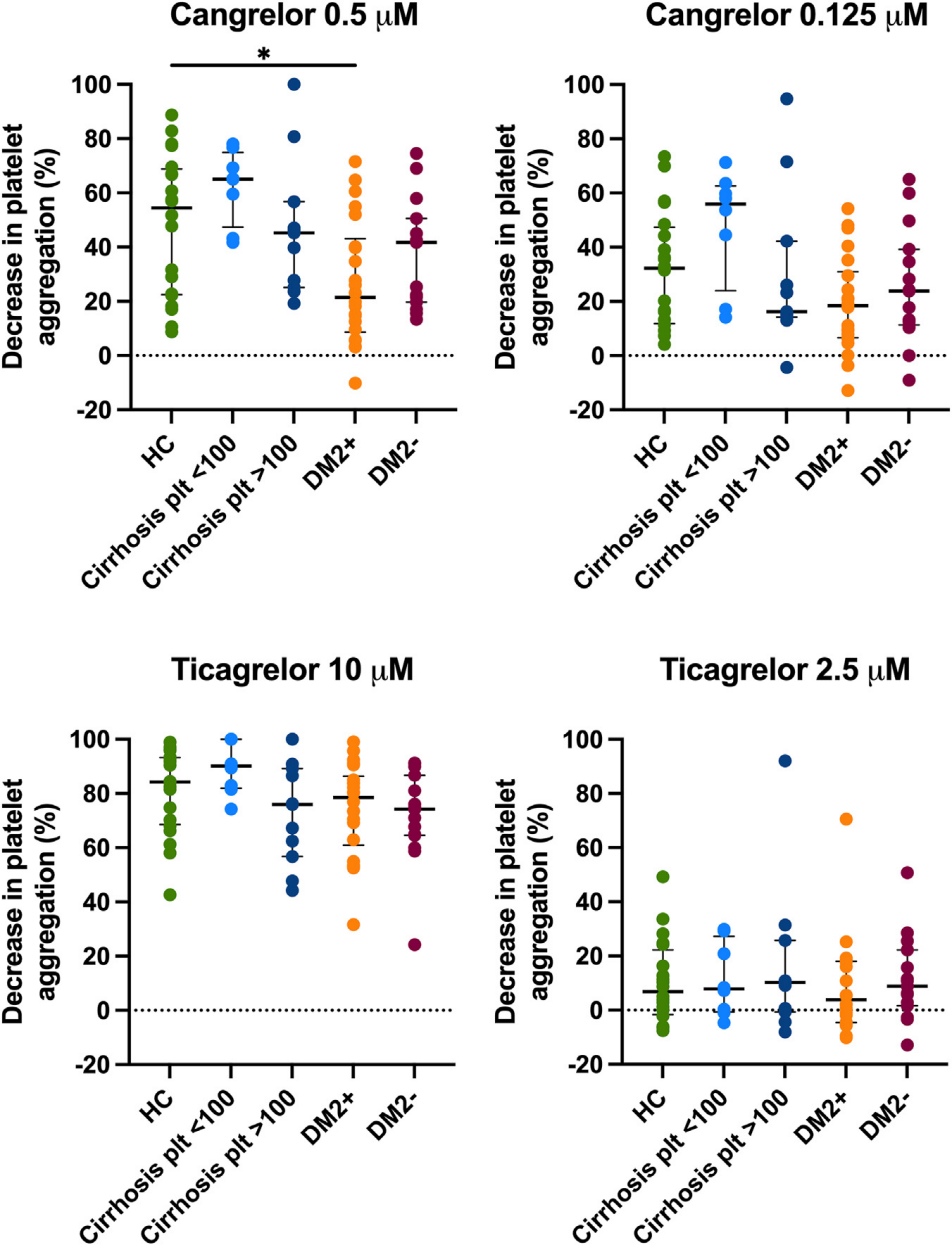
The effect of 0.5 and 0.125 μM of cangrelor and 10 and 2.5 μM of ticagrelor on the 2-MeSADP–induced platelet aggregation at 6 minutes of light transmission aggregometry in HCs, patients with cirrhosis with platelet counts (plt) of below and above 100 × 10^9^/L, patients with type 2 diabetes and steatosis (DM2+), and patients with steatosis only (DM2-). Shown in the figure is the percentual decrease in platelet aggregation in untreated vs treated samples. Bars indicate medians, and error bars indicate IQRs. ∗*P* < .05. 2-MeSADP, 2-methylthioadenosine diphosphate trisodium salt; HC, healthy control.

**Figure 3:**
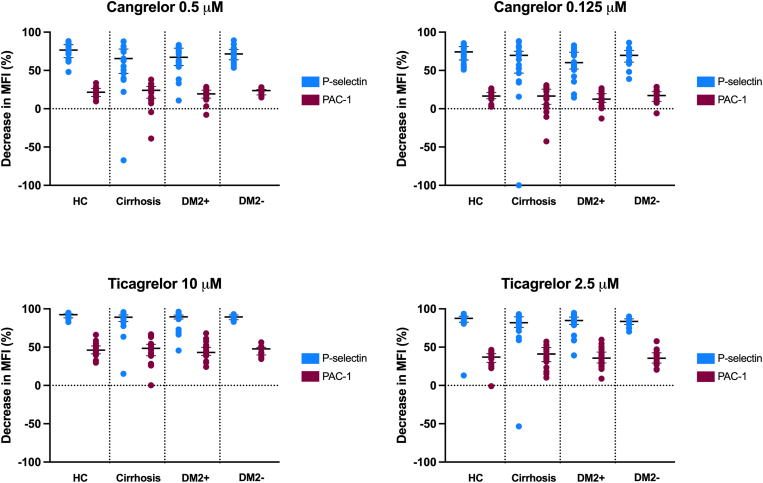
The effect of 0.5 and 0.125 μM of cangrelor and 10 and 2.5 μM of ticagrelor on the 2-methylthioadenosine diphosphate trisodium salt (2-MeSADP)-induced platelet activation in HCs, patients with cirrhosis, patients with type 2 diabetes and steatosis (DM2+), and patients with steatosis only (DM2-). P-selectin and αIIbβ3 expressions were assessed by flow cytometry using anti–P-selectin or the PAC-1 antibody. Shown in the figure is the percentual decrease in MFI in untreated vs treated samples. Bars indicate medians, and error bars indicate IQRs. HC, healthy control; MFI, median fluorescence intensity.

We estimated the antiplatelet effects of cangrelor and ticagrelor by both LTA and flow cytometry. In samples treated with 0.5 μM of cangrelor, 2-MeSADP–induced platelet aggregation decreased to a similar extent in healthy controls, patients with cirrhosis, and patients with steatosis only (Figure 2). In samples of patients with DM2 and steatosis treated with 0.5 μM of cangrelor, the decrease of 2-MesADP–induced platelet aggregation was significantly lower than that in samples of healthy controls (*P* = .028). The lower dose of cangrelor (0.125 μM) however resulted in a similar decrease in 2-MeSADP–induced platelet aggregation across all groups. In samples treated with either 10 or 2.5 μM of ticagrelor, 2-MeSADP–induced platelet aggregation decreased to a similar extent in all groups. The decrease of P-selectin and PAC-1 expression in samples treated with either cangrelor or ticagrelor was similar in all groups regardless of the dose of the drug (Figure 3).

Taken together, in our patient group with MASLD-associated cirrhosis, we found that rates of aspirin resistance were similar to those in healthy controls. In contrast, patients with DM2 and steatosis and patients with steatosis only had higher rates of aspirin resistance than healthy controls. Aspirin resistance suffers from a lack of a standardized definition, and the pathophysiology remains poorly understood. Particularly in patients with DM2, the notion of increased risk of aspirin resistance has been widely accepted over the past decades [7,14,20,21]. However, although our results indeed show an increased rate of aspirin resistance in samples of patients with DM2, we found a similar rate of aspirin resistance in samples of patients without DM2, but with steatosis. The mechanism involved in the process of aspirin resistance may therefore be associated with hepatic steatosis (but not fibrosis) rather than insulin resistance.

Although the *in vitro* potency of cangrelor as measured by LTA was similar in samples of patients with MASLD-associated cirrhosis and healthy controls, we observed a significantly lower antiplatelet effect in samples of patients with DM2 and steatosis. To our knowledge, this is the first study that demonstrates a decreased potency of cangrelor in any specific patient group [18]. Interestingly, these LTA results are not mirrored by our flow cytometry results. This poor correlation between platelet function tests to evaluate antiplatelet drug efficacy has been described previously [22,23], and its clinical relevance remains understudied to this day. Despite this discrepancy, the consequences of a potentially decreased potency warrant additional study, especially since cangrelor is increasingly used to bridge oral P2Y_12_ inhibitors after cardiac interventions [24]. In contrast, we did not find an altered *in vitro* potency of ticagrelor in samples of any of the patient groups compared with samples of healthy controls. The results of this study suggest that ticagrelor may be an adequate antiplatelet agent for use in patients with cirrhosis in clinical practice.

To the best of our knowledge, this is the first study that assessed the *in vitro* potency of antiplatelet drugs in patients with various stages of MASLD, including MASLD-associated cirrhosis. This study therefore adds to the limited knowledge on the efficacy of antiplatelet drugs in patients who have previously been excluded from any phase III and IV trials on these drugs. In particular, our data on patients with MASLD-associated cirrhosis are relevant given the alterations in platelet count and functionality in these patients. Given the altered potency of anticoagulant drugs in patients with MASLD-associated cirrhosis [13,25], studies on potential potency changes in antiplatelet drugs in these patients are of interest.

Limitations of this study include the limited sample size, the lack of biopsy-proven diagnosis of hepatic steatosis at the time of inclusion of patients, the choice of our control group (mainly hospital staff, which may have resulted in differences in socioeconomic status compared to our patient groups), the absence of data on reasons the screened candidates were excluded from our study (due to eligibility screening performed by their primary caregiver prior to contact with the research team), and the *in vitro* nature of the study. Although a biopsy to assess the degree of hepatic steatosis remains the gold standard to diagnose MASLD, the burden of this procedure is substantial and may not balance the benefits for the patients included in this study. Novel techniques such as the FibroScan liver stiffness measurement and CAP are however increasingly used to assess liver disease and aid clinical decision-making, and provide a sufficient estimation of liver fibrosis and steatosis. Importantly, as this study involves *in vitro* addition of antiplatelet drugs to the plasma of patients, we could not assess the effect of metabolism and elimination of these drugs. Additionally, clopidogrel—one of the most used antiplatelet drugs in the clinical setting—is an inactive prodrug that requires hepatic bioactivation [26], and could therefore not be studied in an *in vitro* setting. Nonetheless, this study does show the antiplatelet effects in a controlled setting, and assesses the direct effects of various antiplatelet drugs on platelet aggregation and activation. Naturally, future *in vivo* studies are needed to further assess the efficacy and safety of these drugs in a clinical setting.

In conclusion, we found a similar *in vitro* potency of aspirin, cangrelor, and ticagrelor in samples of patients with MASLD-associated cirrhosis to that in samples of healthy controls. In samples of patients with DM2 and steatosis or patients with steatosis only, the rate of aspirin resistance was higher than in the samples of healthy controls. Additionally, in samples of patients with DM2 and steatosis, the potency of 0.5-μM cangrelor is significantly lower than that in healthy controls as measured by LTA. Future studies are needed to assess the *in vivo* potency of these drugs in patients with MASLD.
