# Supplementary material for: Patients with metabolic dysfunction–associated steatotic liver disease have preserved in vitro responses to antiplatelet drugs
Source: Res Pract Thromb Haemost. 2023 Oct 10;7(7):102217. doi: 10.1016/j.rpth.2023.102217 (PMC10704517; doi:10.1016/j.rpth.2023.102217)
Supplement: Supplementary Material [file mmc1.docx]

**Supplementary document 1.**

*FibroScan measurements*

The liver stiffness and CAP were measured using the FibroScan 530 (Echosens, Paris, France) by a single trained clinical investigator. All patients were fasted for at least 3 hours before the measurements. The liver stiffness score (in kPa) was defined by the median of 10 measurements and was considered reliable if the IQR-to-median ratio of the measurements was at least <30% and if a minimum of 10 successful measurements were obtained. The CAP score was defined by the median value (dB/m) of 10 successful measurements. The M-probe was used in the first instance for all patients. If the M probe failed, the XL probe for obese patients was used.

*Blood samples*

Blood samples for platelet analyses were obtained by venipuncture and collected in 3.2% sodium citrate tubes at a blood-to-anticoagulant ratio of 9:1. All analyses were performed within 2 hours of sampling. Samples were centrifuged at 200g and 2000g for 10 minutes at 18°C to obtain platelet-rich plasma (PRP) and platelet-poor plasma (PPP), respectively. The antiplatelets drugs cangrelor (final concentrations 0.5 and 0.125 μM; Sigma Aldrich, Dorset, UK), ticagrelor (final concentrations 10 and 2.5 μM; Sigma Aldrich, Dorset, UK) or aspirin (final concentration 100 μM; Genzyme Europe bv, Amsterdam, the Netherlands) were added to PRP samples or whole blood, and incubated for 5 minutes at 37^0^C prior to analyses.

*Light transmission aggregometry*

Light transmission aggregometry (LTA) was performed on a TA-8V aggregometer (SD-medical, Stago). Aggregation was initiated by either thrombin receptor activator peptide-6 (TRAP-6; final concentration 10μM; Bachem, Bubendorf, Switzerland), 2-Methylthioadenosine diphosphate trisodium salt (2-MeSADP; final concentration 2 μg/mL; Tocris Bioscience, Bristol, UK), cross-linked collagen related peptide (XL-CRP; final concentration 2 μg/mL; CambCol Laboratories, Cambridge, UK), or arachidonic acid (AA; final concentration 1mM; Hyphen Biomed, Neuville-sur-Oise, France). Light transmission was recorded for 6 minutes at a temperature of 37°C and a rotational speed of 1000 rpm. Platelet aggregation was determined as increase of light transmission, and results are expressed as final platelet aggregation at 6 minutes as a percentage relative to PPP (100%) and PRP (0%). Response to cangrelor and ticagrelor was defined as the percentual increase of 2-MeSADP-induced final platelet aggregation at 6 minutes in samples treated with medication compared to that of samples without medication. Aspirin resistance was defined as AA-induced final platelet aggregation at 6 minutes of less than 20% in samples treated with aspirin^1^.

*Flow cytometry*

Platelet activation in whole blood was studied by flow cytometry as described previously^2^. In short, whole blood was diluted 1:3 with HEPES-buffered saline and 5 μl diluted blood was added to a reaction mixture with a total volume of 20 μl. In this reaction mixture, 2 μl FITC-conjugated PAC-1, 1.5 μl PE-conjugated anti-P-selectin and 0.5 μl APC-conjugated anti-CD42b was present together with a platelet activator or vehicle. The reaction mixtures were incubated for exactly 20 min at 37 °C, and fixated by adding 250 μl fixation solution (137 mmol/l NaCl, 2.7 mmol/l KCl, 1.12 mmol/l NaH2PO4, 1.15 mmol/l KH2PO4, 10.2 mmol/l Na2HPO4, 4 mmol/l EDTA, 0.5% formaldehyde). A NovoCyte Quanteon Flow Cytometer (Agilent, Santa Clara, CA, USA) was used to analyze the samples, and all data were processed using Kaluza Analysis V2.1 (Beckman Coulter, Brea, CA, USA). Fluorescent intensity in the FITC gate and PE channels were determined and results are expressed as median fluorescent intensity (MFI) corrected for the MFI of blood to which no platelet agonist was added. Response to cangrelor and ticagrelor was defined as the percentual 2-MeSADP-induced increase of MFI in FITC and PE channels of samples treated with medication compared to that of samples without medication.

1. Gum PA, Kottke-Marchant K, Welsh PA, White J, Topol EJ. A prospective, blinded determination of the natural history of aspirin resistance among stable patients with cardiovascular disease. *J Am Coll Cardiol*. 2003;41:961–965.

2. Huskens D, Li L, Florin L, et al. Flow cytometric analysis of platelet function to improve the recognition of thrombocytopathy. *Thromb Res*. 2020;194:183–189.
