# Supplementary figures and images for: Patients with metabolic dysfunction–associated steatotic liver disease have preserved in vitro responses to antiplatelet drugs
Source: Res Pract Thromb Haemost. 2023 Oct 10;7(7):102217. doi: 10.1016/j.rpth.2023.102217 (PMC10704517; doi:10.1016/j.rpth.2023.102217)

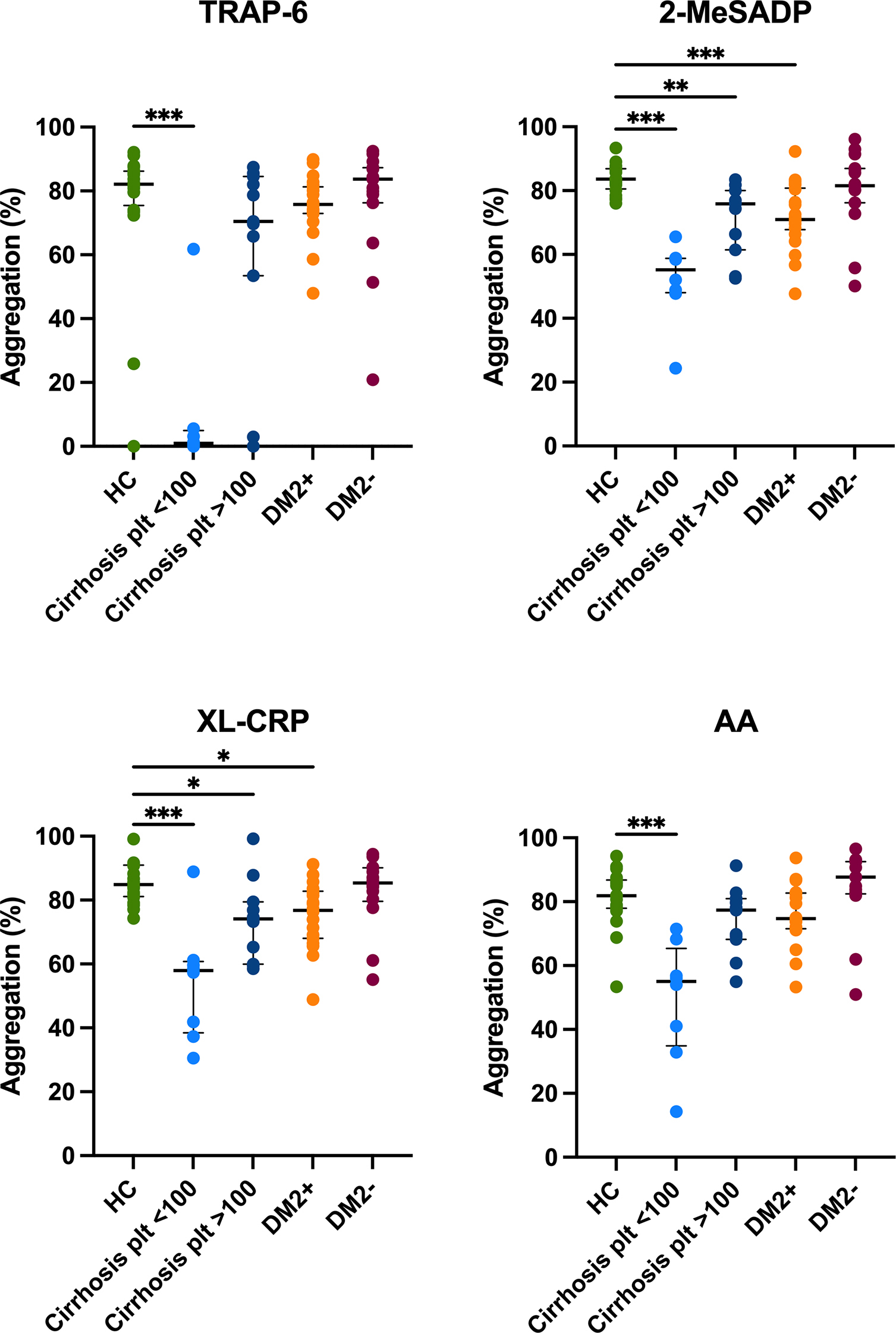

Supplement: Supplementary Figure 1 [file figs1.jpg]

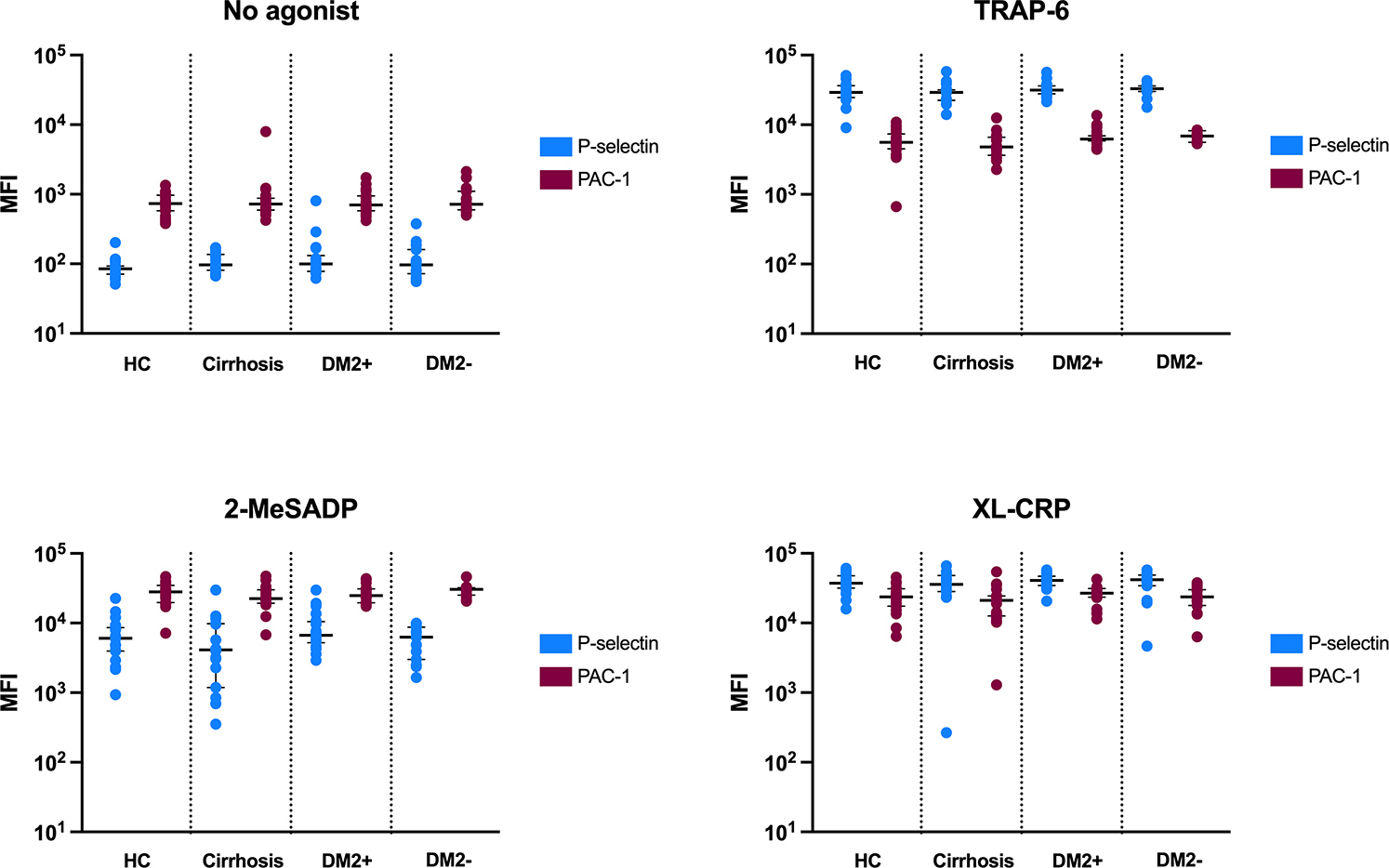

Supplement: Supplementary Figure 2 [file figs2.jpg]
